# Proteomic analysis of small extracellular vesicles from the plasma of patients with hepatocellular carcinoma

**DOI:** 10.1186/s12957-022-02849-y

**Published:** 2022-12-06

**Authors:** Wei Dong, Zeyu Xia, Zehua Chai, Zhidong Qiu, Xuehong Wang, Zebin Yang, Junnan Wang, Tingrui Zhang, Qinqin Zhang, Junfei Jin

**Affiliations:** 1 Xiangya Hospital, Central South University, Changsha, Hunan 410008 China; 2 Guangxi Key Laboratory of Molecular Medicine in Liver Injury and Repair, the Affiliated Hospital of Guilin Medical University, Guilin, 541001 Guangxi China; 3 Guangxi Health Commission Key Laboratory of Basic Research in Sphingolipid Metabolism Related Diseases, the Affiliated Hospital of Guilin Medical University, Guilin, Guangxi 541001 China; 4 China‒USA Lipids in Health and Disease Research Center, Guilin Medical University, Guilin, 541001 Guangxi China; 5 Department of Thyroid and Breast Surgery, Nanxishan Hospital of Guangxi Zhuang Autonomous Region, Guilin, 541002 Guangxi China

**Keywords:** Proteomics, Extracellular vesicles, Hepatocellular carcinoma, Complement

## Abstract

**Purpose:**

Liver cancer is one of the most common tumors with the seventh-highest incidence and the third-highest mortality. Many studies have shown that small extracellular vesicles (sEVs) play an important role in liver cancer. Here, we report comprehensive signatures for sEV proteins from plasma obtained from patients with hepatocellular carcinoma (HCC), which might be valuable for the evaluation and diagnosis of HCC.

**Methods:**

We extracted sEVs from the plasma of controls and patients with HCC. Differentially expressed proteins in the sEVs were analyzed using label-free quantification and bioinformatic analyses. Western blotting (WB) was used to validate the abovementioned sEV proteins.

**Results:**

Proteomic analysis was performed for plasma sEVs from 21 patients with HCC and 15 controls. Among the 335 identified proteins in our study, 27 were significantly dysregulated, including 13 upregulated proteins that were involved predominantly in the complement cascade (complement C1Q subcomponent subunit B (C1QB), complement C1Q subcomponent subunit C (C1QC), C4B-binding protein alpha chain (C4BPA), and C4B-binding protein beta chain (C4BPB)) and the coagulation cascade (F13B, fibrinogen alpha chain (FGA), fibrinogen beta chain (FGB), and fibrinogen gamma chain (FGG)). We verified increased levels of the C1QB, C1QC, C4BPA, and C4BPB proteins in the plasma sEVs from patients with HCC in both the discovery cohort and validation cohort.

**Conclusions:**

The complement cascade in sEVs was significantly involved in HCC progression. C1QB, C1QC, C4BPA, and C4BPB were highly abundant in the plasma sEVs from patients with HCC and might represent molecular signatures.

**Supplementary Information:**

The online version contains supplementary material available at 10.1186/s12957-022-02849-y.

## Introduction

Liver cancer is one of the most common tumors, as liver cancer has the sixth highest incidence and the third highest mortality according to the Global Cancer Statistics 2020 [1]. More than 0.9 million new liver cancer cases were documented in 2018, of which 0.41 million were in China, and 0.83 million deaths occurred, of which 0.39 million were in China. The Global Burden of Disease Study 2019 revealed that China had the highest burden of liver cancer worldwide [2]. Preliminary screening and liquid biopsy of HCC have relied on serum alpha-fetoprotein (AFP) levels. However, due to the low sensitivity of AFP, its levels are frequently normal in patients with HCC and limit the clinical diagnosis, which leads to the delay in early treatment [3]. Thus, new liquid biopsy markers for detection and determining the prognosis are urgently needed.

The small extracellular vesicle (sEV) is a single-bilayer vesicle with a diameter ranging from 30 to 200 nm that originally buds at the plasma and endosomal membranes [4, 5]. sEVs act as not only “garbage bags” but also novel forms of intercellular communication to regulate receiving cells [6, 7]. Although body fluids contain diverse sEVs from multiple cell types, abundant and specific compounds, such as lipids, nucleic acids, and proteins, are closely related to pathological conditions [3]. As a common clinical sample, body fluids are tested for progression, diagnosis, and therapy assessment; however, many potential molecules are difficult to enrich and preserve in body fluids, such as plasma, serum, urine, and saliva. Due to their microstructures, sEVs are stable in body fluids to protect the contents from degradation or destruction. Moreover, the sEVs may be enriched for a trace quantity analysis. Therefore, sEVs are very attractive for liquid biopsy [8].

In recent years, accumulating evidence has suggested that sEVs play important roles in chronic hepatitis, cirrhosis, and liver cancer [9–11]. sEVs from HCC transport LOXL4 and activate the FAK/Src pathway to facilitate the migration of HCC cells [12]. In addition, sEVs from HCC cells deliver LOXL4 to HUVECs and promote angiogenesis through a paracrine mechanism. The sEVs originating from the plasma of patients with HCC contain abundant SMAD3. Moreover, the SMAD3 content in sEVs is positively correlated with the stage of HCC [13].

Due to the close relationship between sEVs and HCC, we aimed to explore potential biomarkers from plasma sEVs for the evaluation of HCC. In this study, by comparing the protein expression among the groups of control subjects and patients with HCC, we comprehensively identified differentially expressed proteins from sEVs using a label-free quantification method. Then, we verified the diagnostic candidates for HCC in the independent validation cohort by performing western blotting.

## Materials and methods

### Plasma sample collection

Plasma samples from controls and patients with HCC were collected at the Affiliated Hospital of Guilin Medical University (Guangxi, China) after written informed consent was obtained from the patients and their families, according to a protocol (#YJSL2021129) approved by the Institutional Review Board of the Affiliated Hospital of Guilin Medical University (Guangxi, China). Twenty-eight patients whose confirmed pathological diagnosis was HCC and 22 control patients without liver cancer. The discovery cohort included 21 patients with HCC and 15 controls. The validation cohort included 7 patients with HCC and 7 controls. All blood samples were collected into EDTA anticoagulant tubes. Briefly, the initial blood samples were sequentially centrifuged at 500 × *g* for 5 min and 2000 × *g* for 20 min at 4 °C to remove the cells and cell debris. The supernatant was then aliquoted into a 2-ml centrifuge tube and stored at − 80 °C.

### Cell line

The HepG2 cell line was used in this study. HepG2 cells were obtained from the American Type Culture Collection. HepG2 cells were cultured in DMEM (Gibco) supplemented with 10% FBS (Gibco) and 1% penicillin/streptomycin at 37 °C with 5% CO_2_.

### Plasma sEV isolation

The clinical blood samples were collected and sequentially centrifuged at 500 × *g* for 5 min and 2000 × *g* for 20 min at 4℃ to remove the cells and cell debris. Then, the supernatants were harvested and centrifuged at 10,000 × *g* for 20 min and subsequently at 12,000 × *g* for 15 min at 4 °C to harvest the supernatants. Moderate exosome isolation reagents (RiboBio, China) with polyethylene glycol (PEG) for precipitation were added to the supernatants. The mixtures were thoroughly shaken and incubated for 30 min at 4 °C. Finally, the mixtures were centrifuged at 15,000 × *g* for 2 min at 4 °C, after which the supernatants were discarded. The pellets were resuspended in 200 μl of PBS and stored at − 80 °C.

### Nanoparticle tracking analysis (NTA)

The size of resuspended sEVs was measured using a NanoSight NS300 instrument (Malvern PANalytical, UK). The distribution and concentration of acquired sEVs were determined and analyzed using NTA software (version 3.1, NanoSight).

### Protein analysis using western blotting

The extracted sEVs were lysed in RIPA buffer (Solarbio, China), boiled, and loaded onto a 10% Tris–Gly gel. The separated proteins were transferred to polyvinylidene difluoride membranes, which were subsequently blocked with 5% skim milk in TBST for 60 min at room temperature. After three washes, the membrane was incubated overnight at 4 °C with primary antibodies against CD63 (ab134045, Abcam), CD81 (ab109201, Abcam), TSG101 (DF8427, Affinity Biosciences), Calnexin (610,523, Becton Dickinson Company), complement C1Q subcomponent subunit B (C1QB; 16919-1-AP, Protein Tech), complement C1Q subcomponent subunit C (C1QC; 16889-1-AP, Proteintech), C4B-binding protein alpha chain (C4BPA; 11819-1-AP, Proteintech), and C4B-binding protein beta chain (C4BPB; 15837-1-AP, Proteintech). After washing, the membrane was incubated for 60 min at room temperature with an HRP-conjugated secondary antibody (Ray, China) and then detected using a Super ECL detection reagent (Yeasen, China). The immunoreactive bands were visualized using a Tanon Automatic Chemiluminescence and Fluorescence Image Analysis System (Tanon, China).

### Transmission electron microscopy (TEM)

Briefly, 5 μl of each sample were placed on Formvar/carbon-coated TEM grids for 3 min at room temperature. The excess fluid at the edge was blotted with filter paper. After rinsing with PBS, the samples were negatively stained with phosphotungstic acid and allowed to dry naturally for 5 min. TEM images of sEVs were captured using a JEM-1200EX TEM (JEOL, Tokyo, Japan).

### Sample preparation for liquid chromatography-tandem mass spectrometry (LC‒MS/MS)

The proteins in sEVs were extracted by lysis by SDT buffer (4% SDS, 100 mM Tris–HCl, and 1 mM dithiothreitol, pH 7.6). The protein concentration was quantified using a BCA Protein Assay Kit. Two hundred micrograms of protein were mixed with 30 μl of SDT buffer (4% SDS, 100 mM dithiothreitol, and 150 mM Tris–HCl pH 8.0). Then, the samples were ultrafiltrated to remove the detergent dithiothreitol (DTT) and the other low molecular weight components in UA buffer (8 M urea and 150 mM Tris–HCl, pH 8.0). The samples were incubated with 100 μl of iodoacetamide (100 Mm iodoacetamide in UA buffer) for 30 min in the dark to block cysteine residues and subsequently digested with 4 μg of trypsin (Promega) in 25 mM NH_4_HCO_3_ buffer overnight at 37 °C. The digested peptides were filtered and collected after washing with 100 μl of UA buffer and 100 μl of 25 mM NH_4_HCO_3_ buffer. The peptides in each sample were desalted on C18 cartridges (Empore SPE Cartridges C18, bed I.D. 7 mm, volume 3 ml, Sigma), concentrated, and reconstituted in 40 µl of 0.1% formic acid (FA). The peptide content was estimated by measuring the absorbance at 280 nm under ultraviolet light.

### LC‒MS/MS

The LC‒MS/MS analysis was performed using a Q Exactive mass spectrometer (Thermo Fisher Scientific) coupled to an Easy-Nlc system (Thermo Fisher Scientific) for 90 min. The peptide mixtures in 0.1% FA were loaded onto a reverse-phase trap column (Thermo Scientific Acclaim PepMap100, 100 μm *2 cm, nanoViper C18), separated using a linear gradient of buffer B (84% acetonitrile and 0.1% FA) at a 300-nL/min flow rate controlled by IntelliFlow technology through a 10-cm-long reverse-phase analytical column (75 μm, 3-μm particle size, C18; Thermo Scientific Easy Column). The mass spectrometer was operated in positive ion mode. Survey MS spectra ranging from m/z 300 to 1800 were acquired at a resolution of 70,000 at m/z 200 using the data-dependent mode to dynamically choose the top 10 most abundant precursor ions for HCD fragmentation (17,500 at m/z 200) with an automatic gain control (AGC) target of 3 × 10^6^ and dynamic exclusion duration of 40.0 s. The normalized collision energy was set to 30 eV, and the underfill ratio was 0.1%. The instrument was operated in peptide recognition mode.

The MS raw data for each sample were combined and searched against the public database UniProt (http://www.uniprot.org/) using MaxQuant 1.5.3.17 software for identification and quantitation. The indexes for MaxQuant identification and quantitation were as follows: two maximum missed cleavages with the trypsin enzyme, carbamidomethylation of cysteine as the fixed modification, and oxidation of methionine as the variable modification. We included contaminants and used the razor and unique peptides for protein quantification with a false discovery rate of peptides and proteins less than 1% to acquire credible identifications of proteins.

The quantitative LC–MS/MS analysis includes the Andromeda score distribution, mass error distribution, and ratio distribution. The criteria for protein identification were a peptide that is unique to a group of highly similar protein sequences (protein group). The criteria for protein contamination were based on the peptide contamination library, which contained the common contaminating protein sequences, such as keratins, bovine serum albumin, and trypsin. These detected contaminants were excluded from further analysis.

### Bioinformatic analysis

#### Domain annotation

InterProScan software was applied to search for protein sequences and align identified protein domain signatures from the InterPro member database Pfam (http://www.pfam.org/).

#### Gene Ontology (GO) annotation

NCBI BLAST + client software and InterProScan were applied to query the selected differentially expressed protein sequences and identify homologous sequences. The Blast2GO program was applied to map GO terms and annotate sequences. The GO annotation results were plotted using R scripts.

#### KEGG functional annotation

Following GO annotation steps, sequence alignment was performed against the Kyoto Encyclopedia of Genes and Genomes (KEGG) database (http://geneontology.org/) to retrieve the KEGG orthologous pathways. Based on their ortholog information, the related KEGG pathway map was constructed.

#### Enrichment analysis

Based on the background dataset of the total quantified proteins, an enrichment analysis was performed using Fisher’s exact test for the *p value*, which was successively adjusted using the Benjamini–Hochberg correction with the false discovery rate (FDR). The functional categories and pathways were significantly enriched and evaluated only if the adjusted *p value* was less than 0.05.

### Statistics

All data were analyzed with GraphPad Prism version 8, SPSS 19.0, ImageJ, or R software. Depending on the data, Student’s *t* test or Fisher’s exact probability test was used to compare differences, and *p* < 0.05 was considered significant. The statistical tests, *p value*s, and adjusted *p value*s for each experiment are shown in the figure legends.

## Results

### Characterization of plasma sEVs

**Fig. 1:**
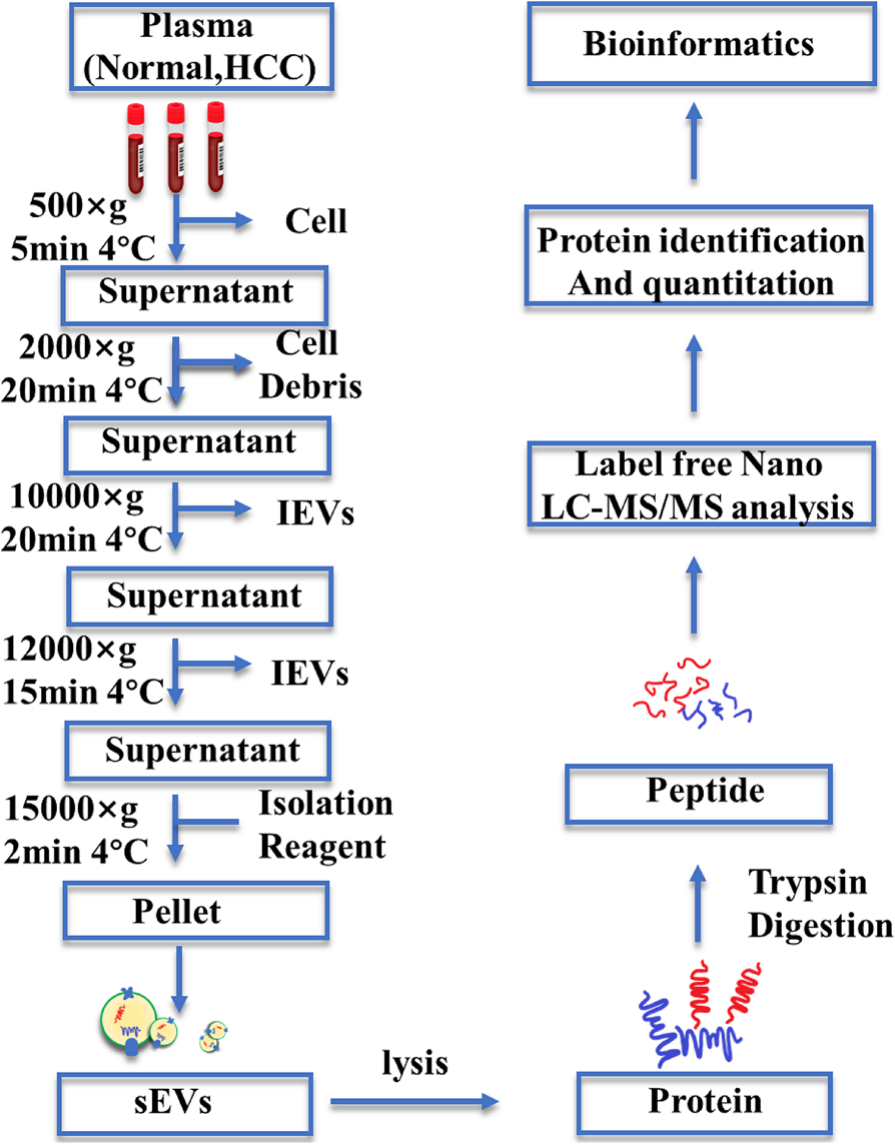
The strategy for sEV isolation by PEG precipitation and bioinformatics analysis

**Fig. 2:**
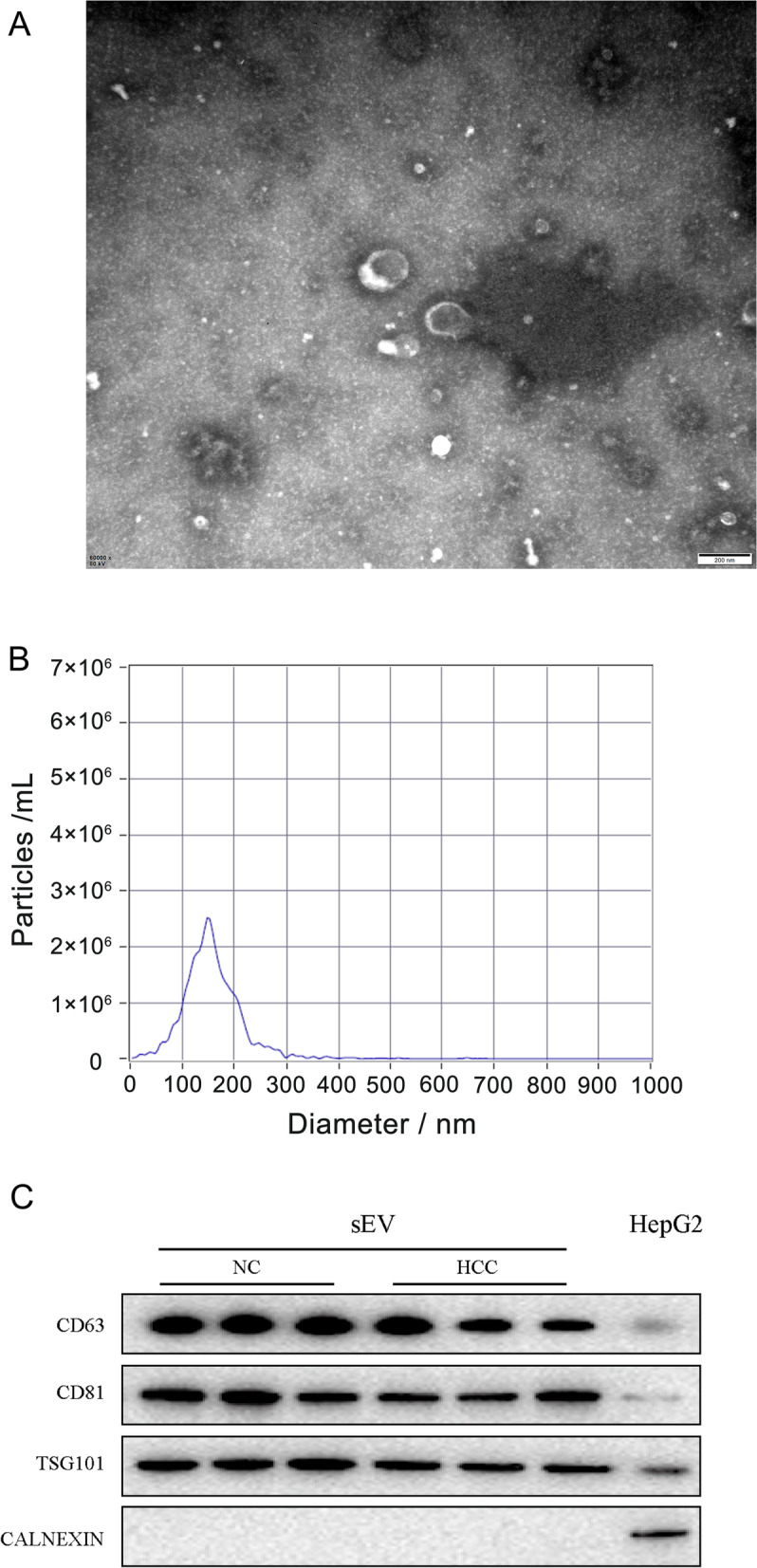
sEV characterization and verification. **A** Representative electron microscopy image of plasma sEVs. Scale bar: 200 nm. **B** The size and concentration distribution of the sEVs in plasma detected using NTA. The particle size distribution of sEVs mainly ranged from 17.5 to 300 nm and peaked at 147.5 nm. **C** The protein expression of three positive markers and one negative marker of sEVs was detected in the sEVs-NC, sEVs-HCC, and cell lysate groups. The protein samples from sEVs extracted from the plasma of control patients and patients with HCC were labeled “sEVs-NC” and “sEVs-HCC,” respectively. The protein sample of the “Cell Lysate” group was extracted from HepG2 cells without any treatment. All samples were normalized by determining the concentration using the BCA assay

We extracted plasma sEVs from 21 patients and 15 controls with an sEV extraction kit (Ribo Bio, China) using polyethylene glycol (PEG) precipitation according to the workflow shown in Fig. 1. The extracted sEVs were confirmed using TEM, which detected their round and cup-like spherical shapes (Fig. 2A), and NTA, which analyzed their diameter and distribution. The average size of the sEVs was 147.5 nm, ranging from 17.5 to 300 nm (Fig. 2B). Compared to the HepG2 cell lysates, the proteins in sEVs were detected using western blotting to determine the presence of three positive markers, CD63, CD81, and TSG101 (Fig. 2C), and the absence of one negative marker, Calnexin (Fig. 2C). As shown in our WB, TEM, and NTA results, the sEV extraction kit we used isolated the sEVs efficiently and accurately.

### Proteomic profiling of plasma sEVs

After identifying the extracted sEVs, a label-free nano-LC–MS/MS analysis was performed to comprehensively analyze the proteins in sEVs and compare them between the normal and HCC groups. Five sEV samples selected randomly and evenly from 15 controls were pooled together to form three new specimens representing the control group, and seven sEV samples from 21 patients with HCC were selected randomly and evenly to form three new specimens representing the HCC group as a method to eliminate the interindividual variability and save money. Three new specimens from the HCC group or control group were subjected to a proteomic analysis. The characteristics of the participants from the HCC group and control group were listed in Supplementary Table 1. The results revealed 850,808 secondary spectral maps, of which 77,850 were matched to the UniProt public database. Using the criteria for protein identification and contamination described in the “Methods” section, 3872 unique peptide segments were identified in a total of 4167 peptide segments, and 281 proteins were quantified among 335 identified proteins.

**Fig. 3:**
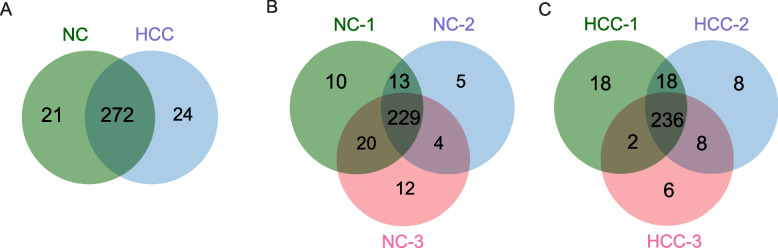
Venn diagrams of the proteins identified in plasma sEVs. **A** Venn diagram of sEV proteins identified in the 3 independent control groups. **B** Venn diagram of sEV proteins identified in the 3 independent HCC groups. **C** Venn diagram of sEV proteins identified in the normal and HCC groups

The overlap of proteins between groups was visualized in a Venn diagram to assess the reproducibility of proteins identified among the groups. Two hundred ninety-three and 296 sEV proteins were identified in the control and HCC groups, respectively (Fig. 3C). A total of 272 proteins were expressed commonly among the control and HCC groups (Fig. 3C). Two hundred twenty-nine (78.2%) proteins in the control group (Fig. 3A) and 236 (79.7%) proteins in the HCC group (Fig. 3B) appeared in all three replicates.

**Fig. 4:**
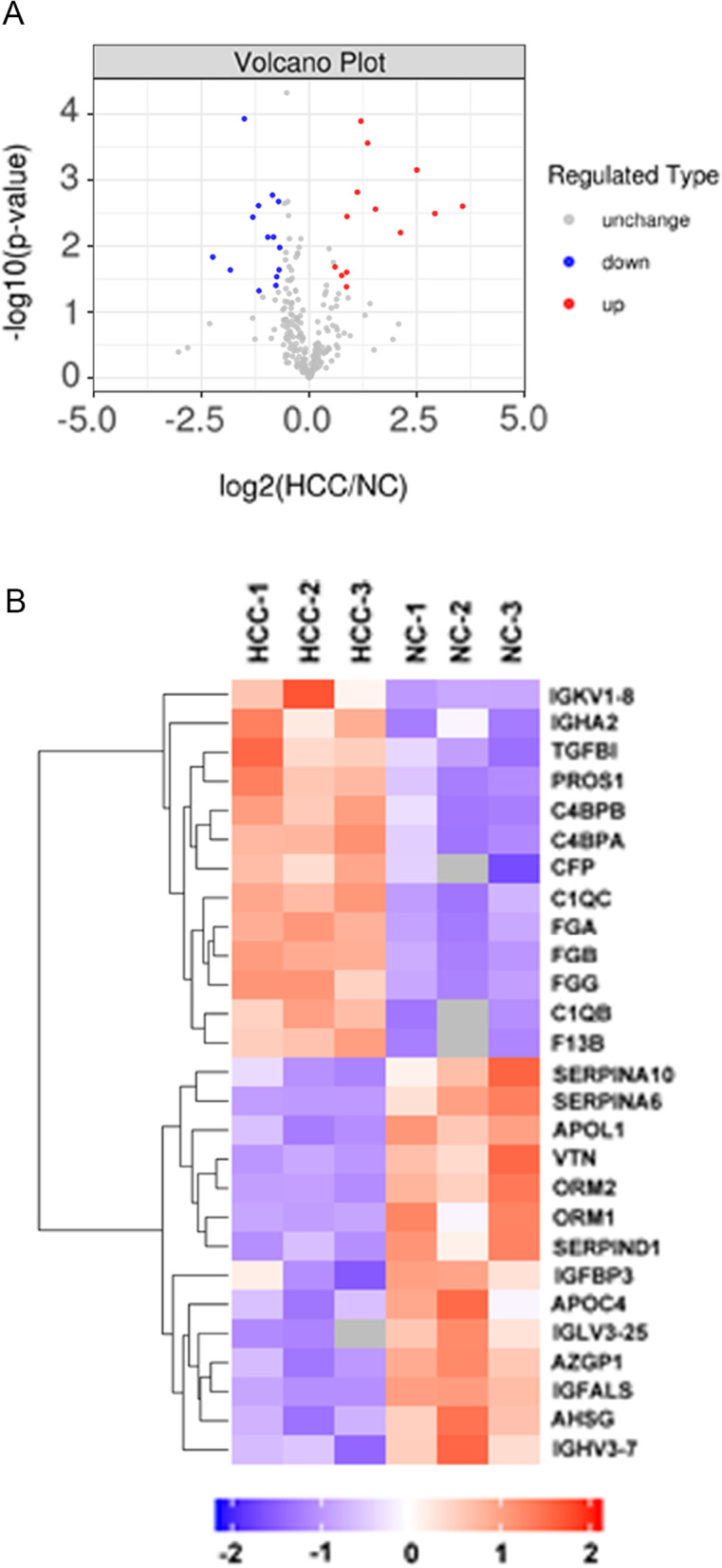
Volcano plot and hierarchical clustering analysis of DEPs in the sEVs from patients with HCC. **A** Volcano plot of protein expression in sEVs from patients with HCC compared to the control group. **B** Heatmap of the hierarchical clustering analysis of DEPs. Each column represents a set of samples (abscissa for sample information), and each row represents a protein (namely, the ordinate for SDEPs). The color scale shown in the map illustrates the relative protein expression: red represents upregulated proteins, blue represents downregulated proteins, and the blanks represent samples for which no quantitative information was available

Compared to the healthy controls, a fold change in HCC sEV proteins ≥ 1.5 or < 0.66 was defined as a differentially expressed protein (DEP) [14, 15], and we identified 54 DEPs in HCC sEVs by performing a quantitative analysis. Among these 54 proteins, the statistical analysis (*p* < 0.05) revealed that only 13 proteins were upregulated and 14 proteins were downregulated in HCC sEVs, as shown in the volcano plot (Fig. 4A). Hierarchical clustering analysis was performed to analyze the patterns of all 27 significantly differentially expressed proteins (SDEPs) in all six samples, and the results are shown in Fig. 4B. In addition, the information related to all 54 DEPs is included in Supplementary Table 2.

### GO and KEGG pathway analyses

We annotated the aforementioned proteins by performing a Gene Ontology (GO) analysis to understand the functions, locations, and biological pathways of the sEV proteins identified in plasma from patients with HCC. GO is a standardized functional classification system that provides a dynamically updated standardized glossary describing the properties of genes and their products in living organisms. The GO functional annotation was divided into three categories: biological process (BP), molecular function (MF), and cellular component (CC).

**Fig. 5:**
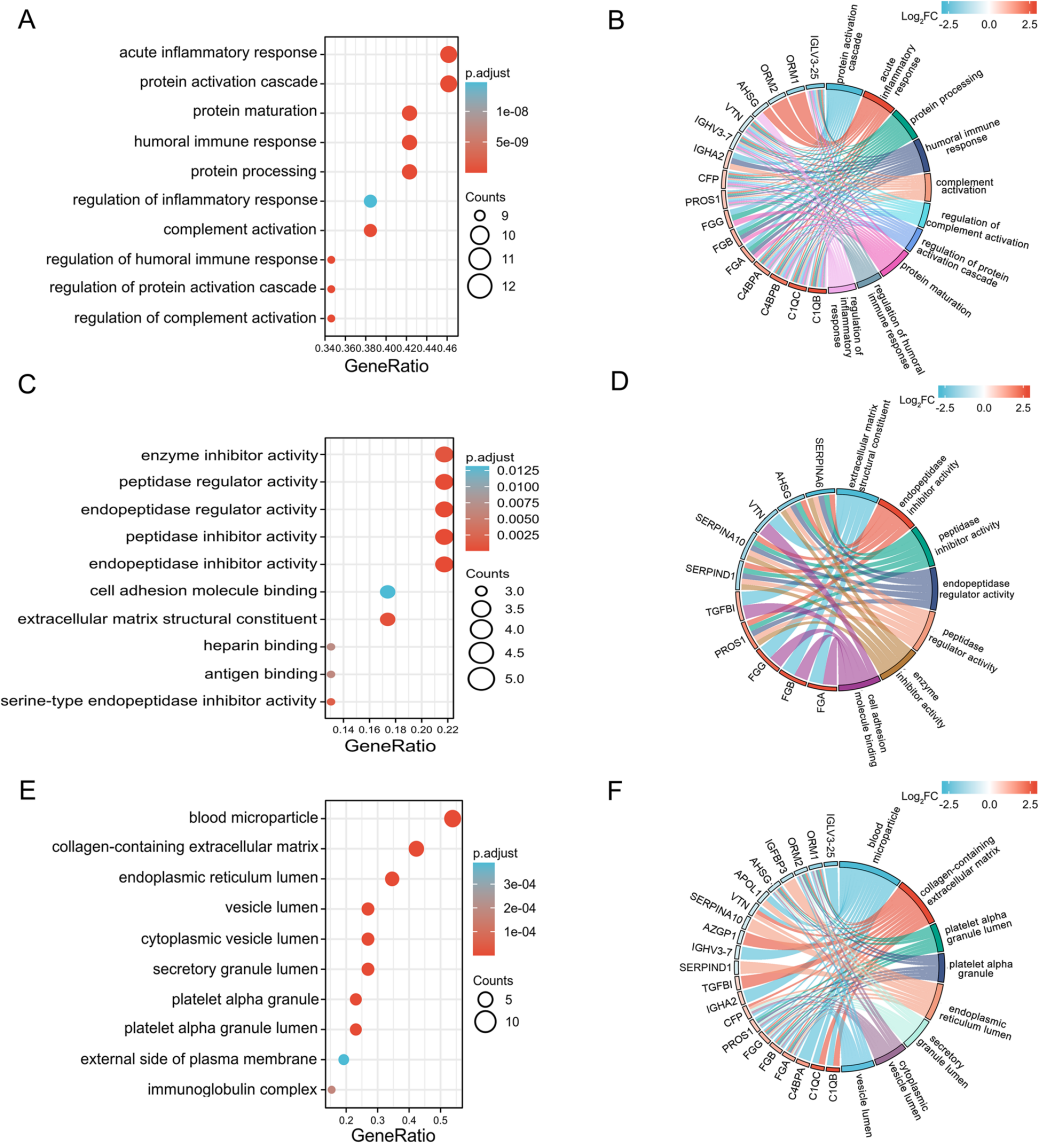
GO analysis of DEPs in the sEVs from patients with HCC. **A** Bubble diagram of the GO functional enrichment analysis of the biological process category. **B** Chord plot of the GO functional enrichment analysis of the biological process category. **C** Bubble diagram of the GO functional enrichment analysis of the molecular function category. **D** Chord plot of the GO functional enrichment analysis of the molecular function category. **E** Bubble diagram of the GO functional enrichment analysis of the cellular component category. **F** Chord plot of the GO functional enrichment analysis of the cellular component category. On the horizontal axis of the bubble diagram, the gene ratio is the ratio of the observed protein count to the background protein count. The color gradient in the bubble diagram represents the adjusted *p value* (-log_10_), and the size of bubbles represents the number of enriched proteins. DEPs are listed on the left of the chord plot and connected to the GO terms. The fold change (Log_2_) is represented by the color indicated in the color bar. The color on the right of the chord plot represents each GO term

We analyzed all 27 SDEPs by determining GO functional annotations compared with the total proteins of reference species and obtained the signature of the difference. The GO analysis of biological processes revealed an enrichment of SDEPs in the “acute inflammatory response,” “protein activation cascade,” “protein maturation,” “humoral immune response,” and “protein processing” (Fig. 5A). A chord diagram revealed the links of SDEPs with biological processes, and the data showed that the majority of SDEPs were involved in the “acute inflammatory response,” including AHSG, C1QB, C1QC, C4BPA, C4BPB, CFP, IGHV3-7, IGLV3-25, ORM1, ORM2, PROS1, and VTN (Fig. 5B). The GO analysis of molecular functions revealed an enrichment of SDEPs involved in “enzyme inhibitor activity,” “peptidase regulator activity,” “endopeptidase regulator activity,” “peptidase inhibitor activity,” and “endopeptidase inhibitor activity” (Fig. 5C). A chord diagram revealed the links of SDEPs with molecular functions, and the data showed that the majority of SDEPs were involved in “endopeptidase inhibitor activity,” including AHSG, PROS1, SERPINA10, SERPINA6, and SERPIND1 (Fig. 5D). The GO analysis of cellular components revealed an enrichment of SDEPs in “blood microparticle,” “collagen-containing extracellular matrix,” “endoplasmic reticulum lumen,” “vesicle lumen,” and “cytoplasmic vesicle lumen” (Fig. 5E). A chord diagram revealed the links of SDEPs with cellular components, and the data showed that the majority of SDEPs were involved in the “collagen-containing extracellular matrix,” including AHSG, AZGP1, C1QB, C1QC, CFP, fibrinogen alpha chain (FGA), fibrinogen beta chain (FGB), fibrinogen gamma chain (FGG), ORM1, ORM2, TGFBI, and VTN (Fig. 5F).

**Fig. 6:**
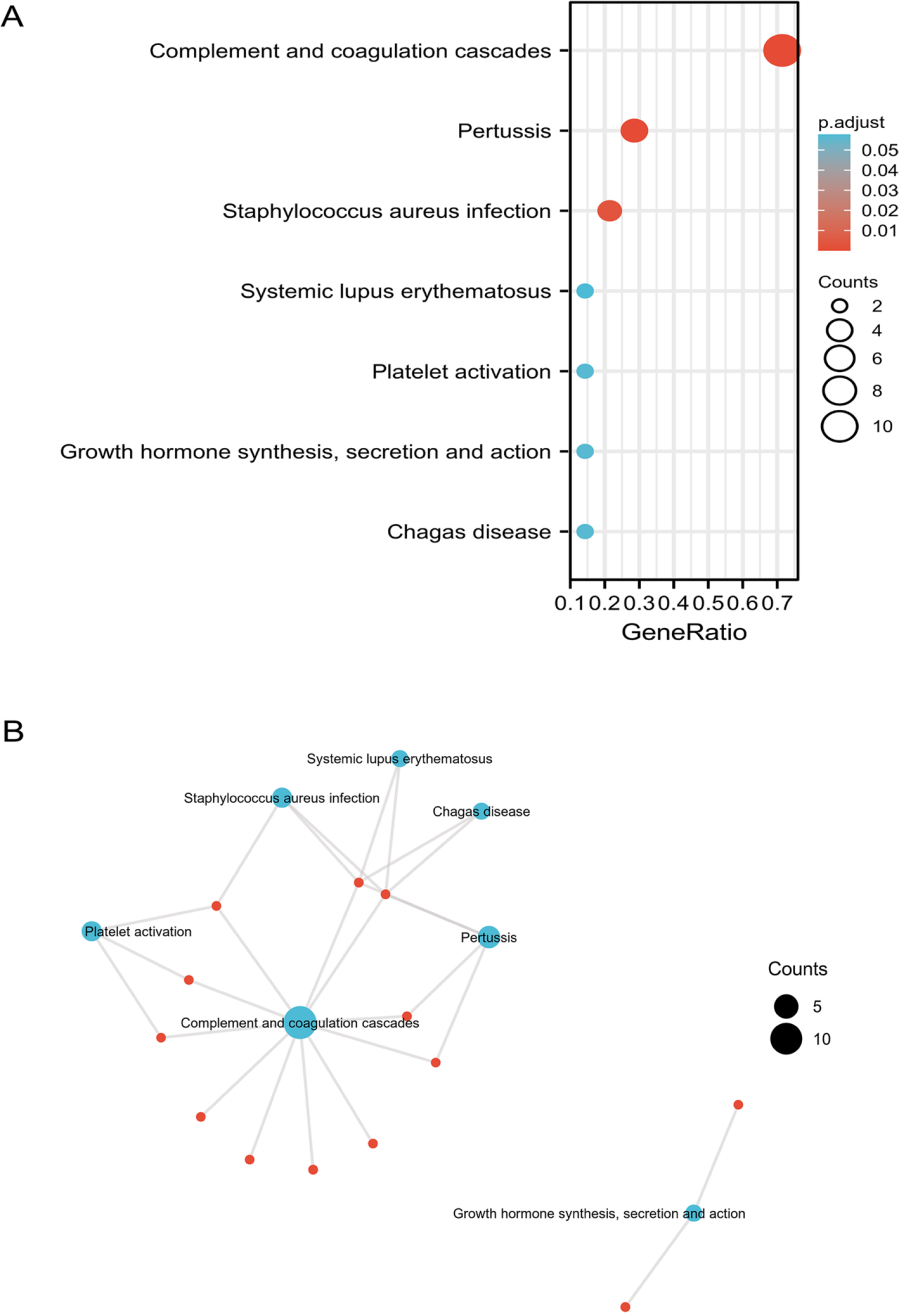
KEGG analysis of DEPs in the sEVs from patients with HCC. **A** Bubble diagram of KEGG pathways. On the horizontal axis of the bubble diagram, the gene ratio is the ratio of the observed protein count to the background protein count. The color gradient in the bubble diagram represents the adjusted *p value* (-log_10_), and the size of bubbles represents the number of enriched proteins. **B** Network of KEGG pathways. Lines represent the relationship between KEGG pathways and DEPs

In addition, SDEPs were analyzed using the KEGG pathway database to predict the related pathways and involved proteins. We found that SDEPs were involved in “complement and coagulation cascades”, “pertussis”, and “Staphylococcus aureus infection” (Fig. 6A). The clustering analysis of KEGG pathways indicated that the SDEPs from HCC plasma sEVs were mainly involved in the complement and coagulation cascade pathways (Fig. 6B), including C1QB, C1QC, C4BPA, C4BPB, F13B, FGA, FGB, FGG, SERPIND1, PROS1, and VTN. Taken together, the results of GO and KEGG analyses suggested that the complement (C1QB, C1QC, C4BPA, and C4BPB) and coagulation (F13B, FGA, FGB, and FGG) pathways are the major pathways in which SDEPs are involved, and the dysregulated proteins of the complement and coagulation pathways may be potential molecular signatures for HCC. Detailed information on the GO and KEGG analyses is provided in Supplementary Table 3.

### Protein network analysis

**Fig. 7:**
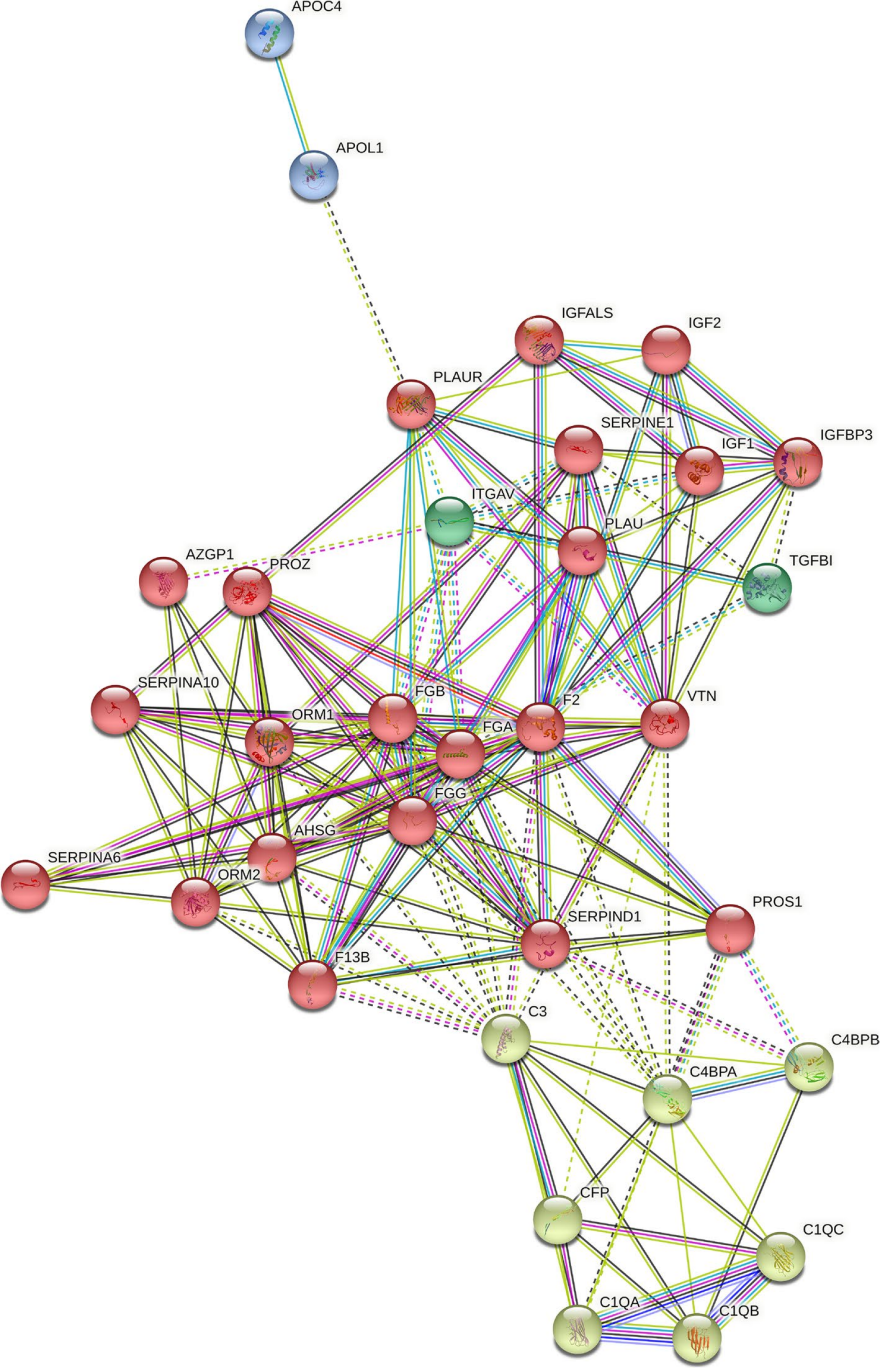
Comprehensive PPI network of DEPs. Interconnecting lines between two proteins indicate the interaction, and the thickness of lines represents the interaction score. The four clusters are marked in different colors

We developed a protein–protein interaction (PPI) network using the Search Tool for Retrieval of Interacting Genes/Proteins (STRING) database to obtain a better understanding of the relationships among these SDEPs and their closely related proteins. Based on previous studies examining these proteins, the relationships among the SDEPs and their closely related proteins were analyzed, and the SDEPs and their closely related proteins were grouped into four clusters, which are labeled in different colors (Fig. 7). Information on these previous studies is included in Supplementary Table 4. In Fig 7, F13B, FGA, FGB, and FGG act as network hubs in the red cluster; C1QB, C1QC, C4BPA, and C4BPB are network hubs in the yellow cluster. These data were consistent with the aforementioned results indicating that F13B, FGA, FGB, FGG, C1QB, C1QC, C4BPA, and C4BPB were upregulated in plasma sEVs from patients with HCC. The raw data from the PPI network are included in Supplementary Tables 5 and 6. Detailed information on the clustering analysis is provided in Supplementary Table 7.

### Validation of complement proteins in sEVs

**Fig. 8:**
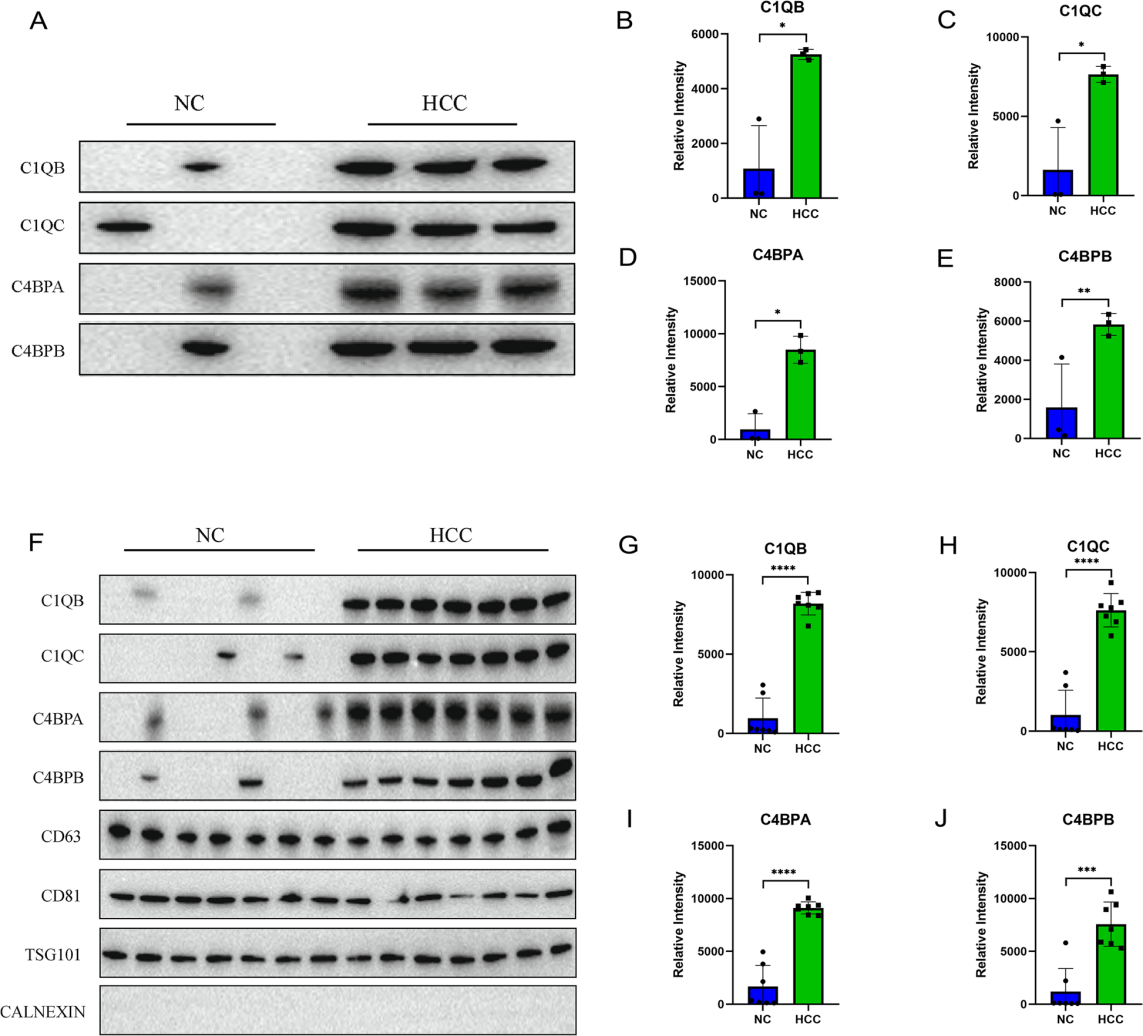
Validation of complement proteins. **A** The protein expression levels of C1QB, C1QC, C4BPA, and C4BPBP in plasma sEVs from the discovery cohort. **B** Column chart showing the comparison of the band densities of C1QB between the control and HCC groups in the discovery cohort. **C** Column chart showing the comparison of the band densities of C1QC between the control and HCC groups in the discovery cohort. **D** Column chart showing the comparison of the band densities of C4BPA between the control and HCC groups in the discovery cohort. **E** Column chart showing the comparison of the band densities of C4BPB between the control and HCC groups in the discovery cohort. **F** The protein expression levels of C1QB, C1QC, C4BPA, and C4BPBP in plasma sEVs from the validation cohort. **G** Column chart showing the comparison of the band densities of C1QB between the control and HCC groups in the validation cohort. **H** Column chart showing the comparison of the band densities of C1QC between the control and HCC groups in the validation cohort. **I** Column chart showing the comparison of the band densities of C4BPA between the control and HCC groups in the validation cohort. **J** Column chart showing the comparison of the band densities of C4BPB between the control and HCC groups in the validation cohort. The left ordinate shows the gray value measured using ImageJ software. *p < 0.05, **p < 0.01, ***p < 0.001, and ****p < 0.0001

We verified the findings from the proteomics analysis by measuring the expression levels of SDEPs involved in the complement cascade using WB in the aforementioned pooled samples. The protein concentrations in sEVs were quantified using the BCA method, and each lane was loaded with the same amount of protein. The SDEPs involved in the complement cascade were increased in plasma sEVs from patients with HCC, including C1QB, C1QC, C4BPA, and C4BPBP (Fig. 8A). The band densities of C1QB (*p* < 0.05), C1QC (*p* < 0.05), C4BPA (*p* < 0.05), and C4BPB (*p* < 0.01) in the HCC group were significantly higher than those in the control group (Fig. 8B–E). These results were consistent with the data from the proteomic analysis.

In addition to the aforementioned pooled samples, we also wanted to detect the protein expression of C1QB, C1QC, C4BPA, and C4BPBP in plasma sEVs from individual patients, and samples from seven patients with HCC and seven normal controls were randomly selected. The results showed increased C1QB, C1QC, C4BPA, and C4BPBP levels in plasma sEVs from patients with HCC (Fig. 8F). The band densities of C1QB (*p* < 0.0001), C1QC (*p* < 0.0001), C4BPA (*p* < 0.0001), and C4BPB (*p* < 0.001) in the HCC group were significantly higher than those in the control group (Fig. 8G–J).

## Discussion

With in-depth research on sEVs, the compositions and functions of sEVs have gradually received attention from researchers in tumor biology and developmental biology. Among the numerous sEV omics studies, most have focused on the contents of miRNAs, lncRNAs, and circRNAs, but few have focused on proteins. Proteomics research on sEVs lags far behind transcriptomics research. Therefore, by applying a proteomics analysis, we aimed to comprehensively detect the DEPs in the sEVs to discover potential biomarkers for HCC. In our study, we launched a systematic approach for analyzing biomarkers in plasma sEVs from patients with HCC, and the following components were included: (1) isolation and identification of sEVs; (2) proteomic profiling of DEPs; (3) domain annotation, enrichment analysis, and network analysis of the DEPs; and (4) validation of the significantly upregulated proteins in the sEVs from patients with HCC.

Our results described the protein signature of plasma sEVs from patients with HCC, including the upregulated pathways of the complement cascade (C1QB, C1QC, C4BPA, and C4BPB) and the coagulation cascade (F13B, FGA, FGB, and FGG). In the coagulation cascade pathway, the mRNA level of FGG and the elevated plasma fibrinogen level are related to the clinical stage, tumor thrombosis, and prognosis of HCC [16, 17]. Asad Uzzaman et al. found that FGA, FGB, and FGG were dysregulated in liver cancer sEVs [18]. However, they focused on fibrinogen and did not further verify the levels of the fibrinogen alpha, beta, and gamma chains in more detail. Their results were consistent with our results, which supported the aforementioned role of fibrinogen in HCC.

As a defensive immune process, the complement system is crucial in several cancers [19]. Recently, accumulating research reported the copresence of extracellular vesicles and the complement system, which indicated a potential link. However, very little information has been published on the relationship between the complement system and extracellular vesicles in patients with HCC. Mao Xiaowen et al. found that complement factor H (CFH) in sEVs derived from HCC promoted tumorigenesis and metastasis [20]. Importantly, in another more recent study, Xie Zhibo et al. showed that the exosome-delivered CD44v6/C1QBP complex promoted a fibrotic liver microenvironment and drove pancreatic cancer liver metastasis [21]. Our findings showed that four components (C1QB, C1QC, C4BPA, and C4BPB) of the complement system were significantly upregulated in the sEVs from the HCC group compared to the control group, which may be a novel discovery in HCC. The classical pathway of complement activation is initiated by C1Q, which is a crucial pattern recognition molecule of the C1 complex and includes three subcomponents (C1QA, C1QB, and C1QC). The C1 complex cleaves C4 into two products, C4A and C4B. As a key component of the C3 and C5 convertases, C4B is essential for the propagation of the classical complement pathway and has many cofactors, such as C4BPA and C4BPB. As negative complement regulators, C4BPA and C4BPB hydrolyze C4B and dissociate the C4B2A complex to terminate complement activation. We speculate that C1QB, C1QC, C4BPA, and C4BPB are involved in the processing of HCC and may modulate the microenvironment in the liver. Although C4BPA and C4BPB appear to play opposite roles to that of C1QB and C1QC, several factors might explain this opposite result. The activation of the complement system has a dual function in development and implications for oncogenesis. On the one hand, as a defensive immune process, complement activation plays an essential role in the immune-mediated killing of tumor cells. However, complement activation promotes inflammation and amplifies tissue injury, which is correlated with tumor progression. Moreover, some complement factors are involved in the immune escape of tumors. As shown in previous studies, C1Q acts in the tumor microenvironment as a cancer-promoting factor [22, 23] and stimulates the β-catenin pathway in liver tumors [24]. Therefore, previous studies provide a plausible explanation for the upregulation of C1QB and C1QC in sEVs, which may be a novel mechanism by which complement components regulate the liver microenvironment. In contrast, the upregulation of C4BP, an inactivator of C4B, correlates with HCC [25, 26]. HCC cells might be protected from complement attack by upregulating C4BPA through binding to the transcription factor SP1 [25], suggesting that C4BPA plays an important role in immune escape. Together, our results were fairly well explained. Based on our results and the cell-to-cell communication mediated by sEVs in HCC, we speculate that the liver microenvironment might be modified by liver tumors via specific cargo in sEVs, and the main cargo is C1QB, C1QC, C4BPA, and C4BPB from the complement system. However, due to the limited sample numbers, this finding and our hypothesis require validation in a larger number of cases with more information on tumor staging and prognosis. Despite the limitations, our research reveals the dysregulated components of the complement system in sEVs from HCC to further support previous studies and provides comprehensive and novel insights into the proteins present in sEVs from patients with HCC.

In addition, we identified 24 HCC-specific proteins that only appeared in sEVs from the HCC group but not in the control group. Among them, only 2 proteins, namely coagulation factor XIII A chain (F13A1) and collectin-11 (COLEC11), were present in all three samples from the HCC group. F13A1 is widely regarded as a cancer-related gene and FDA-approved drug target that is involved in lung cancer [27, 28], oral squamous cell carcinoma [29], and colorectal cancer [30], consistent with our results. Interestingly, COLEC11 has rarely been investigated in oncological studies. Yu Bin et al. established a genomic-clinicopathologic nomogram with a 9-gene-based prognostic index. As one of these 9 genes, dysregulated COLEC11 was related to the early recurrence of HCC after R0 resection [31]. However, the underlying mechanism of dysregulated COLEC11 remains poorly understood. Our results provide an insight into the possible mechanism by which COLEC11 regulates the early recurrence of hepatocellular carcinoma via plasma sEVs. However, this hypothesis requires more experiments for verification. Finally, our study is limited by the relatively small sample size and mixed samples in LC–MS/MS analysis, and therefore, we could not analyze the relationships between SDEPs in sEVs and tumor characteristics including tumor grade, tumor size, microvascular invasion, and TNM staging.

In summary, our study discovered a novel protein signature comprising four upregulated components (C1QB, C1QC, C4BPA, and C4BPB) of the complement system in plasma sEVs from patients with HCC. These factors might be used for noninvasive diagnosis and monitoring tumor progression. This finding has been confirmed by previous studies and our experiments. Our data provide useful insights into the crosstalk between extracellular vesicles and the complement system in HCC.

## Conclusions

Differential and multivariate proteomics analyses of the plasma sEVs from patients with HCC indicated that sEV proteins were significantly related to HCC. Our results described the signature of plasma sEVs from HCC and the upregulated pathway of complement cascades (C1QB, C1QC, C4BPA, and C4BPB) and the coagulation cascade (F13B, FGA, FGB, and FGG), which might be the major factors contributing to the classification of HCC. We also verified the upregulated expression of C1QB, C1QC, C4BPA, and C4BPB. Taken together, these data suggested that an sEV analysis is a valid approach for the evaluation of HCC and that C1QB, C1QC, C4BPA, and C4BPB might be potential molecular signatures.

## Supplementary Information

**Additional file 1.** The general characteristics of our participants.

**Additional file 2.** The information and the statistical results for all DEPs in sEVs.

**Additional file 3.** Data from the GO and KEGG analyses.

**Additional file 4.** The reference publications for the PPI network in the STRING database.

**Additional file 5.** The list of relevant proteins in the PPI network.

**Additional file 6.** The interaction scores in the PPI network.

**Additional file 7.** The data of the clustering analysis based on the STRING database.

**Additional file 8.** The raw data of band density in the discovery cohort.

**Additional file 9.** The raw data of band density in the validation cohort.

## Data Availability

All authors will have full access to the final trial dataset. The data supporting the conclusions of this article is included within the article and its additional file. The full protocol and participant-level dataset will be made available upon request if agreed upon by the corresponding author.

## Abbreviations

HCC: Hepatocellular carcinoma
AFP: Alpha-fetoprotein
sEV: Small extracellular vesicle
C1QB: Complement C1Q subcomponent subunit B
C1QC: Complement C1Q subcomponent subunit C
C4BPA: C4B-binding protein alpha chain
C4BPB: C4B-binding protein beta chain
FGA: Fibrinogen alpha chain
FGB: Fibrinogen beta chain
FGG: Fibrinogen gamma chain
F13A: Coagulation factor XIII A chain
F13B: Coagulation factor XIII B chain
LC–MS/MS: Liquid chromatography-tandem mass spectrometry
CC: Cellular component
MF: Molecular function
GO: Gene Ontology
KEGG: Kyoto Encyclopedia of Genes and Genomes
