# Supplementary material for: Proteomic analysis of small extracellular vesicles from the plasma of patients with hepatocellular carcinoma
Source: World J Surg Oncol. 2022 Dec 6;20:387. doi: 10.1186/s12957-022-02849-y (PMC9724420; doi:10.1186/s12957-022-02849-y)
Supplement: Supplementary file 1 — Additional file 1. The general characteristics of our participants. [file 12957_2022_2849_MOESM1_ESM.pdf]

Supplementary Table 1:

Variables	NC	HCC	P-value
**Age**			0.2537
**≤50**	7	14	
**＞50**	15	14	
**Sex**			0.7608
**male**	14	20	
**female**	8	8	
**HBsAg(ng/mL)**			0.5355
**≤ 0.5**	8	7	
**> 0.5**	14	21	
**ALT(U/L)**			0.2533
**≤ 40**	10	18	
**> 40**	12	10	
**AST(U/L)**			0.3926
**≤ 40**	10	17	
**> 40**	12	11	
**Cirrhosis**			0.1501
**YES**	11	20	
**NO**	11	8	
**Smoking**			0.7527
**YES**	7	7	
**NO**	15	21	
